# COVID-19 patients with increasing age experience differential time to initial medical care and severity of symptoms

**DOI:** 10.1017/S095026882100234X

**Published:** 2021-10-22

**Authors:** J. Mancilla-Galindo, A. Kammar-García, A. Martínez-Esteban, H. D. Meza-Comparán, J. Mancilla-Ramírez, N. Galindo-Sevilla

**Affiliations:** 1 Unidad de Investigación UNAM-INC, Instituto Nacional de Cardiología Ignacio Chávez, Mexico City, Mexico; 2 Sección de Estudios de Posgrado e Investigación, Escuela Superior de Medicina, Instituto Politécnico Nacional, Mexico City, Mexico; 3 Dirección de Investigación, Instituto Nacional de Geriatría, Mexico City, Mexico; 4 Facultad de Medicina, Universidad Nacional Autónoma de México, Mexico City, Mexico; 5 Hospital de la Mujer, Secretaría de Salud, Mexico City, Mexico; 6 Departamento de Infectología e Inmunología, Instituto Nacional de Perinatología, Secretaría de Salud, Mexico City, Mexico

**Keywords:** Ageing, coronavirus, COVID-19, epidemiology, pandemic

## Abstract

We conducted a retrospective observational study in patients with laboratory-confirmed coronavirus disease (COVID-19) who received medical care in 688 COVID-19 ambulatory units and hospitals in Mexico City between 24 February 2020 and 24 December 2020, to study if the elderly seek medical care later than younger patients and their severity of symptoms at initial medical evaluation. Patients were categorised into eight groups (<20, 20–29, 30–39, 40–49, 50–59, 60–69, 70–79 and ≥80 years). Symptoms at initial evaluation were classified according to a previously validated classification into respiratory and non-respiratory symptoms. Comparisons between time from symptom onset to medical care for every age category were performed through variance analyses. Logistic regression models were applied to determine the risk of presenting symptoms of severity according to age, and mortality risk according to delays in medical care. In total, 286 020 patients were included (mean age: 42.8, s.d.: 16.8 years; 50.4% were women). Mean time from symptom onset to medical care was 4.04 (s.d.: 3.6) days and increased with older age categories (*P* < 0.0001). Mortality risk increased by 6.4% for each day of delay in medical care from symptom onset. The risk of presenting with the symptoms of severity was greater with increasing age categories. In conclusion, COVID-19 patients with increasing ages tend to seek medical care later, with higher rates of symptoms of severity at initial presentation in both ambulatory units and hospitals.

## Introduction

Delayed hospitalisation is an independent risk factor for death, intensive care unit (ICU) admission and invasive mechanical ventilation (IMV) in patients with coronavirus disease (COVID-19) [1]. Older adults with COVID-19 who are hospitalised have been noted to have different severe acute respiratory syndrome coronavirus 2 (SARS-CoV-2) viral kinetics than younger patients, with a slower decline of viral load after its peak value, which is an independent risk factor for death [2]. Furthermore, older patients experience delayed times from symptom onset to a positive reverse-transcriptase polymerase chain reaction (RT-PCR) test result for SARS-CoV-2 [3], as well as longer incubation periods [4, 5].

The finding that older adults have longer incubation periods is thought to be due to slower and less robust immune responses with advancing age [5]. Further complicating this, older adults often have atypical clinical presentations when diagnosed with COVID-19 [6]. Altogether, these differences of SARS-CoV-2 infection in older adults could be related to delayed diagnosis, treatment and suboptimal public health measures to limit spread of the disease.

Few studies to date have evaluated delays in medical care of patients with COVID-19 [7, 8]. Unfortunately, most studies evaluating the impact of COVID-19 in the elderly are limited due to small sample sizes or unrepresentative populations of the whole spectrum of disease (i.e. hospitalised-only patients).

In this study, we sought to study if the elderly seek medical care later than younger patients and their severity of symptoms at initial medical evaluation in a population-based cohort from Mexico City of ambulatory and hospitalised patients diagnosed with COVID-19.

## Methods

### Study design

We conducted a retrospective observational study in patients who received medical care for suspected COVID-19 in 688 registered and accredited COVID-19 ambulatory units and hospitals in Mexico City between 24 February 2020 and 24 December 2020. In this study, 935 204 patients were considered for eligibility. All patients with a positive RT-PCR for SARS-CoV-2 were included to maximise the power and generalisability of the study.

### Source of data

We used the COVID-19 open dataset available in Mexico City Government's Open Data platform [9], which is collected and updated daily by the Secretariat of Health of Mexico City. Patients meeting criteria of suspected COVID-19 case have been included in this dataset starting on 24 February 2020 when the first suspected cases arrived in Mexico. Detailed diagnostic criteria for inclusion in this dataset, as well as details on diagnostic testing, follow-up mechanisms and variables included have been described elsewhere [10].

### Management of variables

Patients were grouped into the following age categories: <20, 20–29, 30–39, 40–49, 50–59, 60–69, 70–79 and ≥80 years. History of exposition to confirmed COVID-19 cases within the last 7 days was determined through anamnesis and categorised into yes, no or unknown. Time from symptom onset to medical care was defined as the difference in days between the date of appearance of the first symptom and the date of ambulatory care or hospitalisation. A variable of critical patients was created by grouping patients requiring IMV and/or admission to an ICU. Time to hospitalisation in the subgroup of critical patients was calculated to distinguish if patients requiring critical care sought medical care later than hospitalised patients not requiring critical care. To assess severity of symptoms at initial medical evaluation, we used a previously created classification of symptoms used in Mexican patients with COVID-19 that distinguishes non-respiratory symptoms from respiratory symptoms, the latter of which have been associated with the lowest survival probability and greatest mortality risk [11]. Respiratory symptoms included one or more of the following symptoms: dyspnoea, polypnoea, cyanosis, fever or cough. Patients with non-respiratory symptoms were those who had the absence of respiratory symptoms and one or more of the following: headache, myalgias, arthralgias, general deterioration, abdominal pain, chest pain, conjunctivitis, irritability or vomiting.

### Statistical analysis

Descriptive data were calculated and are provided as frequencies and percentages for qualitative variables and mean with standard deviation (s.d.) or standard error (s.e.), and median for quantitative variables. Normality of quantitative data was corroborated with asymmetry (±0.5) and kurtosis (±2). Comparisons between time from symptom onset to medical care, as well as for number of symptoms for every age category, were performed through variance analyses of one factor alongside the Welch correction test. The Games-Howell *post hoc* analysis was used to determine the differences between pairwise comparisons; the category of 40–49 years was set as the reference since mean age of most studies included in systematic reviews evaluating incubation periods and time to medical care fall in this category [12, 13]. The mean time from symptom onset to medical care for every age category was graphed alongside their 95% confidence interval (95% CI) to aid interpretation.

Two logistic regression analyses were applied to determine the risk of presenting with respiratory symptoms according to age; the variable of age was used as a quantitative variable in one analysis and as a categorical variable in a subsequent analysis in which the category of <20 years was set as the reference since patients in this category have the lowest mortality risk. The results of the first analysis were plotted as odds ratio (OR) and their 95% CI for every year. For the second analysis, OR and their corresponding 95% CI were represented in a forest plot for every category of age. A third logistic regression analysis was applied to determine mortality risk according to delays in medical care, considering time from symptom onset to medical care as a continuous variable.

Weekly testing rates for every age category were calculated for every 100 000 inhabitants in Mexico City in different age categories and graphed for every week in the year 2020. The total number of inhabitants in Mexico City for every 10-year age category were obtained from the 2020 Population and Housing Census [14].

A two-sided *P* value <0.05 was used to define statistical significance. Analyses and figures were created with SPSS software v.21, R software v.3.4.1 and GraphPad Prism v.9.0.

## Results

Out of 935 204 patients assessed for eligibility, 571 866 with a negative RT-PCR for SARS-CoV-2 and 77 318 with inconclusive results were excluded. In total, 286 020 patients with a positive RT-PCR for SARS-CoV-2 were included for analysis. Of these, 50.4% (*n* = 144 228) were women. Patients from a wide range of ages were included (0–120 years), with a mean age of 42.8 (s.d.: 16.8) years and a median of 42.0 years. Children and adolescents (<18 years) constituted 5.2% (*n* = 14 755) of patients, with a mean age of 11.4 (s.d.: 4.9) years. Pregnant women (mean age: 29.2 (s.d.: 7.1) years) represented 0.4% (*n* = 1106) of all patients and 0.8% of all women. Indigenous people represented 0.4% (*n* = 1185) of patients in our cohort. The baseline and follow-up characteristics of patients in every age category, including the total count of respiratory and non-respiratory symptoms, are provided in Table 1.

**Table 1.** Baseline and follow-up characteristics of patients according to their age category

	Total	<20 years	20–29 years	30–39 years	40–49 years	50–59 years	60–69 years	70–79 years	≥80 years
Number of patients, *n* (%)	286 020 (100)	19 654 (6.9)	47 942 (16.8)	60 534 (21.2)	59 376 (20.8)	50 114 (17.5)	29 533 (10.3)	13 486 (4.7)	5381 (1.9)
Sex									
Women, *n* (%)	144 228 (50.4)	9694 (49.3)	24 730 (51.6)	30 618 (50.6)	30 209 (50.9)	25 574 (51.0)	14 322 (48.5)	6381 (47.3)	2700 (50.2)
Men, *n* (%)	141 792 (49.6)	9960 (50.7)	23 212 (48.4)	29 916 (49.4)	29 167 (49.1)	24 540 (49.0)	15 211 (51.5)	7105 (52.7)	2681 (49.8)
Age	42.84 (16.88)	13.2 (5.26)	25.16 (2.76)	34.43 (2.88)	44.59 (2.86)	54.21 (2.85)	63.90 (2.83)	73.78 (2.80)	84.89 (4.72)
Days from symptom onset to medical care	4.04 (3.62)	3.2 (3.25)	3.74 (3.43)	3.89 (3.47)	4.12 (3.61)	4.28 (3.75)	4.51 (3.88)	4.63 (3.99)	4.40 (3.88)
Type of medical care									
Ambulatory, *n* (%)	247 025 (86.4)	18 947 (96.4)	46 706 (97.4)	57 126 (94.4)	53 024 (89.3)	41 005 (81.8)	20 302 (68.7)	7403 (54.9)	2512 (46.7)
Hospitalised, *n* (%)	38 995 (13.6)	707 (3.6)	1236 (2.6)	3408 (5.6)	6352 (10.7)	9109 (18.2)	9231 (31.3)	6083 (45.1)	2869 (53.3)
ICU^a^, *n* (%)	3154 (8.1)	134 (19.0)	123 (10.0)	259 (7.6)	510 (8.0)	728 (8.0)	735 (8.0)	481 (7.9)	184 (6.4)
IMV^a^, *n* (%)	7892 (20.2)	82 (11.6)	159 (12.9)	460 (13.5)	1134 (17.9)	1905 (20.9)	2116 (22.9)	1460 (24.0)	576 (20.1)
Critical^a^, *n* (%)	8485 (21.8)	155 (21.9)	189 (15.3)	528 (15.5)	1233 (19.4)	2008 (22.0)	2236 (24.2)	1529 (25.1)	607 (21.2)
Comorbidities									
Diabetes, *n* (%)	33 103 (11.6)	105 (0.5)	517 (1.1)	1887 (3.1)	5867 (9.9)	9889 (19.7)	8726 (29.5)	4551 (33.7)	1561 (29.0)
COPD, *n* (%)	2803 (1.0)	22 (0.1)	57 (0.1)	113 (0.2)	240 (0.4)	485 (1.0)	746 (2.5)	676 (5.0)	464 (8.6)
Asthma, *n* (%)	6081 (2.1)	553 (2.8)	1219 (2.5)	1393 (2.3)	1258 (2.1)	914 (1.8)	489 (1.7)	189 (1.4)	66 (1.2)
Immunosuppression, *n* (%)	2796 (1.0)	179 (0.9)	181 (0.4)	355 (0.6)	504 (0.8)	629 (1.3)	504 (1.7)	334 (2.5)	110 (2.0)
Hypertension, *n* (%)	41 754 (14.6)	95 (0.5)	707 (1.5)	2581 (4.3)	6782 (11.4)	11 663 (23.3)	10 754 (36.4)	6386 (47.4)	2786 (51.8)
HIV infection, *n* (%)	1088 (0.4)	18 (0.1)	160 (0.3)	295 (0.5)	219 (0.4)	204 (0.4)	126 (0.4)	48 (0.4)	18 (0.3)
CVD, *n* (%)	4574 (1.6)	112 (0.6)	208 (0.4)	350 (0.6)	612 (1.0)	938 (1.9)	1039 (3.5)	816 (6.1)	499 (9.3)
Obesity, *n* (%)	41 053 (14.4)	796 (4.1)	4663 (9.7)	8753 (14.5)	10 315 (17.4)	8974 (17.9)	4999 (16.9)	1986 (14.7)	567 (10.5)
CKD, *n* (%)	3338 (1.2)	68 (0.3)	198 (0.4)	355 (0.6)	493 (0.8)	737 (1.5)	786 (2.7)	501 (3.7)	200 (3.7)
Smoker, *n* (%)	30 820 (10.8)	582 (3.0)	6711 (14.0)	8018 (13.2)	6524 (11.0)	4605 (9.2)	2666 (9.0)	1243 (9.2)	471 (8.8)
Count of symptoms									
Total	3.99 (2.81)	2.67 (2.46)	3.64 (2.68)	4.04 (2.73)	4.13 (2.78)	4.16 (2.87)	4.35 (2.91)	4.64 (2.95)	4.74 (2.90)
Non-respiratory	2.48 (2.01)	1.61 (1.77)	2.31 (1.9)	2.58 (2.01)	2.60 (2.02)	2.56 (2.03)	2.58 (2.01)	2.69 (2.03)	2.69 (1.99)
Respiratory	1.51 (1.16)	1.07 (1.01)	1.32 (1.05)	1.45 (1.08)	1.52 (1.14)	1.59 (1.20)	1.76 (1.26)	1.94 (1.30)	2.05 (1.29)
At least one respiratory symptom, *n* (%)	221 591 (77.5)	12 640 (64.3)	35 864 (74.8)	47 160 (77.9)	46 723 (78.7)	39 438 (78.7)	23 900 (80.9)	11 266 (83.5)	4600 (85.5)
Individual symptoms									
Fever, *n* (%)	144 173 (50.4)	7543 (38.4)	22 036 (46.0)	30 877 (51.0)	31 035 (52.3)	26 056 (52.0)	15 932 (53.9)	7602 (56.4)	3092 (57.5)
Cough, *n* (%)	181 685 (63.5)	9658 (49.1)	29 048 (60.6)	38 650 (63.8)	38 439 (64.7)	32 828 (65.5)	19 950 (67.6)	9412 (69.8)	3700 (68.8)
Sore throat, *n* (%)	112 772 (39.4)	5953 (30.3)	19 328 (40.3)	25 642 (42.4)	24 266 (40.9)	19 772 (39.5)	11 228 (38.0)	4854 (36.0)	1729 (32.1)
Dyspnoea, *n* (%)	71 164 (24.9)	2330 (11.9)	8169 (17.0)	12 032 (19.9)	14 168 (23.9)	14 335 (28.6)	11 038 (37.4)	6251 (46.4)	2841 (52.8)
Irritability, *n* (%)	43 124 (15.1)	2741 (13.9)	7121 (14.9)	9327 (15.4)	9026 (15.2)	7627 (15.2)	4330 (14.7)	2086 (15.5)	866 (16.1)
Diarrhoea, *n* (%)	53 335 (18.6)	2476 (12.6)	9080 (18.9)	12 234 (20.2)	11 367 (19.1)	9260 (18.5)	5362 (18.2)	2522 (18.7)	1034 (19.2)
Chest pain, *n* (%)	65 572 (22.9)	2310 (11.8)	9559 (19.9)	14 166 (23.4)	14 716 (24.8)	12 371 (24.7)	7371 (25.0)	3630 (26.9)	1449 (26.9)
Chills, *n* (%)	89 320 (31.2)	3712 (18.9)	13 938 (29.1)	20 083 (33.2)	19 921 (33.6)	16 430 (32.8)	9340 (31.6)	4243 (31.5)	1653 (30.7)
Headache, *n* (%)	177 903 (62.2)	9521 (48.4)	30 412 (63.4)	40 141 (66.3)	38 389 (64.7)	31 036 (61.9)	17 536 (59.4)	7862 (58.3)	3006 (55.9)
Myalgias, *n* (%)	126 985 (44.4)	4686 (23.8)	19 174 (40.0)	28 337 (46.8)	28 094 (47.3)	23 417 (46.7)	14 106 (47.8)	6585 (48.8)	2586 (48.1)
Arthralgias, *n* (%)	112 932 (39.5)	3787 (19.3)	16 136 (33.7)	24 857 (41.1)	25 326 (42.7)	21 239 (42.4)	12 992 (44.0)	6140 (45.5)	2455 (45.6)
Abrupt deterioration, *n* (%)	104 781 (36.6)	4132 (21.0)	15 036 (31.4)	22 180 (36.6)	22 471 (37.8)	19 396 (38.7)	12 510 (42.4)	6328 (46.9)	2728 (50.7)
Rhinorrhoea, *n* (%)	83 522 (29.2)	5806 (29.5)	16 481 (34.4)	19 608 (32.4)	17 064 (28.7)	13 161 (26.3)	7066 (23.9)	3125 (23.2)	1211 (22.5)
Polypnoea, *n* (%)	25 589 (8.9)	1015 (5.2)	3209 (6.7)	4380 (7.2)	4984 (8.4)	5041 (10.1)	3782 (12.8)	2165 (16.1)	1013 (18.8)
Vomit, *n* (%)	17 258 (6.0)	1043 (5.3)	2782 (5.8)	3492 (5.8)	3488 (5.9)	3080 (6.1)	2002 (6.8)	1004 (7.4)	367 (6.8)
Abdominal pain, *n* (%)	28 632 (10.0)	1611 (8.2)	4608 (9.6)	6072 (10.0)	6080 (10.2)	5059 (10.1)	2989 (10.1)	1590 (11.8)	623 (11.6)
Conjunctivitis, *n* (%)	32 787 (11.5)	1837 (9.3)	6242 (13.0)	8106 (13.4)	7063 (11.9)	5369 (10.7)	2625 (8.9)	1145 (8.5)	400 (7.4)
Cyanosis, *n* (%)	9872 (3.5)	385 (2.0)	1245 (2.6)	1739 (2.9)	1897 (3.2)	1900 (3.8)	1454 (4.9)	843 (6.3)	409 (7.6)
Sudden onset of symptoms, *n* (%)	79 987 (28.0)	4570 (23.3)	12 771 (26.6)	17 247 (28.5)	16 793 (28.3)	14 193 (28.3)	8545 (28.9)	4144 (30.7)	1724 (32.0)
Case-fatality rate	16 114 (5.6)	50 (0.3)	171 (0.4)	637 (1.1)	1799 (3.0)	3446 (6.9)	4551 (15.4)	3566 (26.4)	1894 (35.2)

CKD, chronic kidney disease; COPD, chronic obstructive pulmonary disease; CVD, cardiovascular disease; HIV, human immunodeficiency virus; ICU, intensive care unit; IMV, invasive mechanical ventilation.

Data are presented as mean with s.d., unless otherwise specified.

a Proportion out of hospitalised patients.

Out of all patients, 86.4% (*n* = 247 025) received ambulatory care, whereas 13.6% (*n* = 38 995) were hospitalised. Of the hospitalised patients, 21.8% (*n* = 8485) required critical care, 20.2% (*n* = 7892) underwent IMV and 8.1% (*n* = 3154) were admitted to ICU. The proportion of hospitalised patients requiring critical care increased with older age categories starting from the 20–29 years category. The baseline and follow-up characteristics of patients according to modality of care received (ambulatory, hospitalisation or critical care) are provided in Table 2.

**Table 2.** Baseline and follow-up characteristics of patients according to modality of care received (ambulatory, hospitalisation or critical care)

	Ambulatory	Hospitalised	Critical
Number of patients, *n* (%)	247 025 (86.4)	38 995 (13.6)	8485 (3.0)
Sex			
Women, *n* (%)	129 446 (52.4)	14 782 (37.9)	2772 (32.7)
Men, *n* (%)	117 579 (47.6)	24 213 (62.1)	5713 (67.3)
Age	40.59 (15.87)	57.09 (16.24)	58.64 (15.65)
Days from symptom onset to medical care	3.84 (3.50)	5.32 (4.11)	5.35 (4.15)
Comorbidities			
Diabetes, *n* (%)	21 714 (8.8)	11 389 (29.2)	2713 (32.0)
COPD, *n* (%)	1482 (0.6)	1321 (3.4)	314 (3.7)
Asthma, *n* (%)	5389 (2.2)	692 (1.8)	121 (1.4)
Immunosuppression, *n* (%)	1665 (0.7)	1131 (2.9)	255 (3.0)
Hypertension, *n* (%)	28 782 (11.7)	12 972 (33.3)	3033 (35.7)
HIV infection, *n* (%)	872 (0.4)	216 (0.6)	36 (0.4)
CVD, *n* (%)	2875 (1.2)	1699 (4.4)	395 (4.7)
Obesity, *n* (%)	32 285 (13.1)	8768 (22.5)	2187 (25.8)
CKD, *n* (%)	1486 (0.6)	1852 (4.7)	418 (4.9)
Smoker, *n* (%)	26 766 (10.8)	4054 (10.4)	975 (11.5)
Count of symptoms			
Total	3.65 (2.70)	6.15 (2.51)	6.2 (2.52)
Non-respiratory	2.33 (1.99)	3.43 (1.89)	3.36 (1.92)
Respiratory	1.32 (1.06)	2.72 (1.08)	2.86 (1.04)
At least one respiratory symptom, *n* (%)	183 771 (74.4)	37 820 (97.0)	8337 (98.3)
ICU	0 (0.0)	3154 (8.1)	3154 (37.2)
IMV	0 (0.0)	7892 (20.2)	7892 (93.0)
Case-fatality rate	1497 (0.6)	14 617 (37.5)	6294 (74.2)

CKD, chronic kidney disease; COPD, chronic obstructive pulmonary disease; CVD, cardiovascular disease; HIV, human immunodeficiency virus; ICU, intensive care unit; IMV, invasive mechanical ventilation.

Data are presented as mean with s.d., unless otherwise specified.

**Fig. 1.**
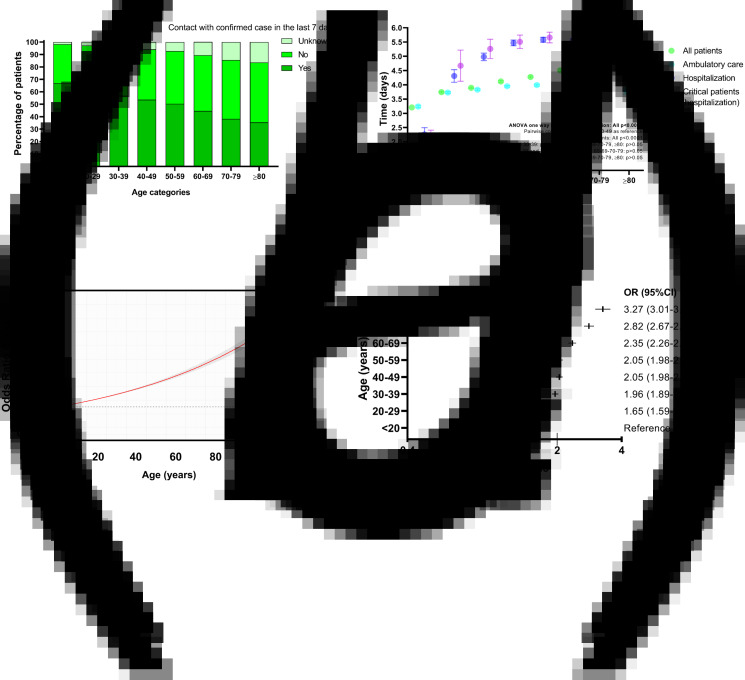
Contact with confirmed COVID-19 cases, time from symptom onset to medical care and risk of having symptoms of severity at initial presentation in laboratory-confirmed COVID-19 patients in Mexico City. (a) Proportion of patients who had contact with a confirmed COVID-19 case in the last 7 days according to age categories. (b) Mean and 95% CI of time from symptom onset to medical care according to age categories. (c) Risk of respiratory symptoms at initial presentation according to age as a continuous variable. (d) Risk of respiratory symptoms at initial presentation according to age categories. OR, odds ratio; 95% CI, 95% confidence interval.

Known contact with a confirmed COVID-19 case within 7 days was more frequent in younger patients than elderly patients (Fig. 1a). The mean time from symptom onset to medical care was 4.04 (s.d.: 3.6) days (3.84 (s.d.: 3.5) days for ambulatory care and 5.32 (s.d.: 4.1) days for hospitalised patients). Significant differences in these times occurred for most age categories (Fig. 1b), whereas trends show that patients in the <20 years category had the shortest time-to-medical attention and for every 10-year increase patients received medical care later up to the 70–79 years category; for patients ≥80 years this tendency was reversed and patients received care earlier with every 10-year increase in age. Mortality risk increased by 6.4% for each day of delay in time to medical care from symptom onset after adjusting for age and sex (*β* = 0.064, OR 1.06, 95% CI 1.06–1.07, *P* < 0.0001).

Patients experienced a mean symptom count of 3.99 (s.d.: 3.8) symptoms. In patients experiencing respiratory and non-respiratory symptoms, the mean count was 1.51 (s.d.: 1.2) and 2.48 (s.d.: 2.0), respectively.

When evaluating the risk of presenting respiratory symptoms with increasing ages, we observed that the odds of presenting respiratory symptoms increased 1.3% (*β* = 0.013, OR 1.013, 95% 1.012–1.013, *P* < 0.0001) for every 1-year increase in age (Fig. 1c). The increase in risk for age as a continuous variable was not strictly log-linear. In the risk analysis according to age categories, all categories were significant risk factors for presenting with respiratory symptoms at initial evaluation when compared with the <20 years reference category, with greater risks occurring with increasing age (Fig. 1d).

**Fig. 2.**
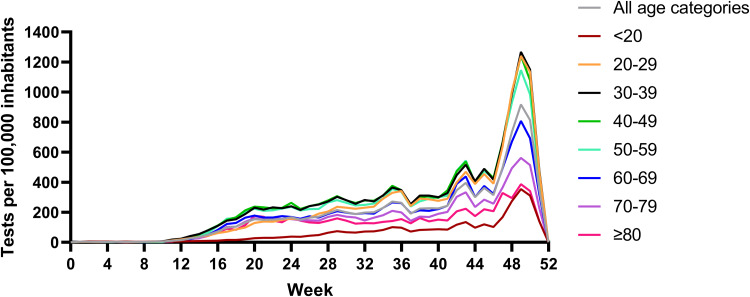
Weekly diagnostic testing rates for SARS-CoV-2 in Mexico City according to age categories during 2020.

The weekly diagnostic testing rate for every age category during the entire study period is shown in Figure 2. Patients with ages between 20 and 59 years had the highest testing rates, whereas patients in the <20 and ≥80 years categories had the lowest testing rates.

## Discussion

Our results indicate that patients in different categories of age tend to experience different times to medical care from symptom onset and varying severity of symptoms at presentation, while also experiencing different recall of known contact with a confirmed COVID-19 case within the prior 7 days, and different testing rates according to age category.

Patients with increasing age sought medical care later in our study, with higher rates of symptoms of severity at initial presentation in both ambulatory and hospitalised patients, although this tendency changed at ages >80 years since patients sought ambulatory or hospital care earlier, having more symptoms of severity at admission. Receiving medical care later was associated with an increase in mortality risk of 6.4% for every 1-day delay which highlights the importance of timeliness of medical care. Differences in time to medical care could be because patients with increasing age were hospitalised more frequently, since patients tended to seek ambulatory care similarly. However, patients in the <20, 20–29 and 30–39 years categories had shorter time to ambulatory care and time to hospitalisation, which could reflect that younger patients seek medical attention earlier despite having lower rates of hospitalisation and respiratory symptoms which are associated with an increased risk of death [11]. Furthermore, patients ≥80 years tended to have shorter time to hospitalisation despite having the highest rate of hospitalisation, as well as a shorter time to ambulatory care, and the highest count of respiratory symptoms at presentation. Similarly, Faes *et al*. report that patients experiencing the largest delay in hospitalisation are those in the 20–60 years category, followed by 60–80 years and those in the >80 years category are hospitalised earlier [8].

It is important to note that, despite age category, patients in Mexico City sought medical care later than what has been described for patients in other countries where patients sought ambulatory care 2.1 (s.d.: 2.65) days after symptom onset [15], which contrasts with patients in our study (3.84 (s.d.: 3.50) days). Compared with other countries, patients who were hospitalised in Mexico City were overall younger and with a mean time to hospitalisation of 5.32 days which was similar to Belgium (5.74 days) and the UK (5.14 days) which had greater proportions of older patients, and longer than Singapore (2.62 days) and Hong Kong (4.41) [7, 8]. Therefore, delayed medical attention in Mexico City could reflect structural deficiencies in the health system which lead patients to seek care later. Alternatively, this could be an idiosyncratic feature of Mexico City's population possibly related to sociocultural and behavioural particularities.

The risk of presenting with respiratory symptoms at the initial evaluation increased with advancing age, although the increase in risk was not log-linear. The risk increased in magnitude with every 10-year increase in age, being highest in the ≥80 years category (OR 3.27, 95% CI 3.01–3.55). This finding could be explained by age-related decline in airway clearance and gradual decrease of cilia and ciliated cells in the airway with ageing [16], alongside altered immune responses to SARS-CoV-2 with increasing age [17].

Previous studies had shown that increasing age is associated with longer incubation periods after SARS-CoV-2 infection [5, 6, 13]. Although we were not able to calculate precise incubation periods for patients in our study, we studied history of contact with a confirmed case within 7 days. For every 10-year increase, patients reported more frequently no contact with confirmed cases in the past 7 days, which could reflect that patients tended to have increasing incubation periods with older ages. However, such pronouncement is only hypothetical and cannot be derived from our results due to the fact that only a 7-day period history of exposition (with significant risk of variable collection and recall bias) was determined.

Importantly, patients in different age categories have had important differences in SARS-CoV-2 testing rates in Mexico City, with the highest testing rates being in young adults and middle-aged adults; the lowest testing rates occurred in the <20 and ≥80 year categories. Although an important increase in testing capacity occurred with the introduction of antigen tests in Mexico City after 28 October 2020 [18], this pattern of testing rates by age category did not change, which suggests that low availability of testing may not determine whether younger people and older adults seek testing or not. Rather, lower testing rates in older adults, children and adolescents could be explained by the tendency to stay at home during the pandemic [19], as well as lower contacts with other people, and lower mobility indexes than young adults and middle-aged adults [20].

Lower testing rates in patients <20 and ≥80 years could have biased our study towards representation of more symptomatic patients who could have sought testing more frequently than less symptomatic patients. However, this is not necessarily true since patients in these two categories had opposite behaviours. Although patients <20 years tended to receive care earlier and with less symptoms of severity, those ≥80 years also sought medical care earlier but with higher rates of symptoms of severity.

Limitations of our study include that we could not directly calculate incubation periods, since we determined history of exposition to confirmed COVID-19 cases through anamnesis, which does not necessarily correlate with the real incubation period, posing a high risk of variable collection and recall bias. Also, our study is limited by the fact that we could not assess disease progression according to clinical criteria since parameters for staging patients are not captured in this dataset, and thus we used severity of symptoms (respiratory symptoms) as a correlate of disease severity at admission since respiratory symptoms are strongly associated with the risk of death and other adverse outcomes. The exclusion of patients with a negative RT-PCR for SARS-CoV-2 could have introduced bias in our study since a negative RT-PCR result does not necessarily imply the absence of the disease and we did not compare characteristics of patients with negative, positive and pending testing results, which have been shown to have different baseline and follow-up characteristics, as well as different associations with mortality risk in a previous study performed in Mexican ambulatory and hospitalised patients [21]. Furthermore, patients <20 and ≥80 years were likely underrepresented in our study due to lower testing rates in these age categories, which compromise validity of our findings. Finally, our study is limited due to its retrospective nature.

The strengths of our study include the large sample size from a population-based cohort of patients evaluated for suspected COVID-19 in 688 ambulatory units and hospitals across Mexico City. Since obtaining and uploading these data occurs prospectively and responsible health authorities are held accountable for this labour, this dataset has nearly complete data. The fact that we used a previously validated categorisation of symptoms to identify patients at risk of death and adverse outcomes further strengthens our study.

Future studies could evaluate disease severity in different age groups to further elucidate if the elderly could have similar patterns of seeking medical attention in populations with distinct characteristics than Mexico City's inhabitants. Such studies should seek to include both ambulatory and hospitalised patients to adequately capture the whole spectrum of COVID-19 disease progression.

## Conclusion

Patients with increasing ages tend to seek medical care later, with higher rates of symptoms of severity at initial presentation in both ambulatory and hospitalised patients, although this tendency is changed at ages >80 years since patients seek care earlier, having more symptoms of severity at admission. Future studies could further characterise delays in medical care experienced by patients with COVID-19, as well as their impact on clinical outcomes and the factors associated with delays in medical care.

## Data Availability

The data that support the findings of this study are openly available in the Open Data Platform of Mexico City's Government at https://datos.cdmx.gob.mx/dataset/base-covid-sinave [9].
